# *Prevotella* in the airway: implications for lung health and pathogen defense mediated by *Prevotella*-host interactions

**DOI:** 10.1128/jb.00228-26

**Published:** 2026-08-12

**Authors:** Sara N. Stoner, Ana Fairbanks-Mahnke, Sarah E. Clark

**Affiliations:** 1 Department of Otolaryngology–Head & Neck Surgery, University of Colorado School of Medicine, Aurora, Colorado, USA; Dartmouth College Geisel School of Medicine, Hanover, New Hampshire, USA

**Keywords:** *Prevotella*, lung microbiome, pneumonia, CF, COPD, bacterial infection

## Abstract

*Prevotella* species are an extremely common and abundant bacteria detected within the low microbial biomass of the lungs and are a core component of the oral microbiome. Clinical studies have drawn associations between *Prevotella* abundance and lung homeostasis, indicating potential relationships between *Prevotella* and lung inflammation, infection defense, and lung function. Across several studies in critically ill patients, the depletion of *Prevotella* and other obligate anaerobes is linked to significantly reduced survival, leading to calls for anaerobe preservation in empiric antibiotic therapy regimens. In recent years, mechanistic studies have provided new information regarding *Prevotella*-host relationships, highlighting several processes by which *Prevotella* exposure activates epithelial, innate, and adaptive immune responses. *Prevotella* species also have direct and indirect effects on important lung bacterial pathogens, including *Streptococcus pneumoniae*, *Staphylococcus aureus*, and *Pseudomonas aeruginosa*, with *Prevotella* species-dependent consequences for pathogen infection and regulation of pathogen-induced inflammation. This review summarizes our current understanding regarding how *Prevotella* regulate lung immune homeostasis, with a discussion of key knowledge gaps necessary for the translation of these insights into new therapeutic approaches to reduce the burden of lung infection and disease.

## INTRODUCTION

Lower respiratory tract infections are a leading cause of death globally, accounting for 2.6 million deaths per year, with bacterial pneumonia contributing to the greatest proportion of this morbidity (1, 2). However, many people are colonized with bacterial lung pathogens such as *Streptococcus pneumoniae* (the pneumococcus) or *Staphylococcus aureus* without developing pneumonia. One of the underlying features contributing to protection against lung infection is the microbiome, through both direct pathogen interference and indirect modulation of host immune homeostasis. In both humans and animal models, microbiome dysbiosis following antibiotic therapy is associated with a period of enhanced susceptibility to lung infection, an effect also observed in germ-free mice (3–7). Microbiome replacement, or reconstitution, reverses this enhanced susceptibility (4, 5). Antibiotic treatment has been shown to suppress Th17 cells in the lung (8)—an immune population that is critical for defense against bacterial pneumonia (9). Chronic lung diseases are associated with substantial shifts in lung microbiome composition, with correlative evidence for relationships between lung microbial populations and lung inflammatory responses, exacerbations, and lung function. Here, we spotlight *Prevotella*, a core component of the oral and lung microbiome. By examining clinical correlations together with *in vivo* and *in vitro* evidence (Table 1), we demonstrate that respiratory tract *Prevotella* have a significant impact on lung immune tone and pulmonary disease outcomes. Furthermore, we posit that *Prevotella* species heterogeneity underlies variable infection and disease associations, demonstrating a need for additional characterization of how distinct *Prevotella* species impact immune regulation and overall pulmonary health.

**TABLE 1:** Key observations on *Prevotella* summarized by prior support in literature

Observation	Clinical support	Murine support	*In vitro* support
*Prevotella* species as an anti-inflammatory or homeostatic organism found in states of health	(10–15)	(16)	(17–20)
Loss of *Prevotella*/commensal anaerobes is associated with dysbiosis and disease	(21–31)		
*Prevotella* species are associated with altered pulmonary disease outcomes	Respiratory failure and hospitalization: (32) NTM and other infections: (33) Asthma: (34–36) Cystic Fibrosis: (37–40) Other: (41–43)		
*Prevotella* species promote host defense and inhibit respiratory pathogen infection		(44–47)	(19, 44, 48)
*Prevotella* species promote Th17 inflammation in the airway	(49)	(50)	
*Prevotella* species shape airway community structure and influence interactions with other microbes	(51)		(48, 52, 53)
The functional consequences of airway *Prevotella* metabolism on lung homeostasis	(54–56)		(57–59)
Select *Prevotella* species enhance secondary bacterial infections		(60, 61)	(53)

## *PREVOTELLA* AS A DEFINITIVE FEATURE OF THE AIRWAY MICROBIOME

While the human lungs were previously thought to be sterile, advances in sequencing and culture methods in recent decades have proven the existence of a limited biomass microbial community in the lower airways, resulting primarily from aspiration of oropharyngeal bacteria including *Prevotella* effectively “seeding” the lower airway (10, 62). *Prevotella* are a core member of the oral microbiome, detected in high abundance across most individuals (11, 63). In the lower airway, *Prevotella* are typically within the top three most abundant bacteria detected (10, 64–66). *Prevotella* are gram-negative obligate anaerobes, suggesting that oxygen gradients are a critical feature of respiratory tract survival. *Prevotella* survival in oxygen-rich niches is thought to be facilitated by the presence of facultative or aerobic species, which deplete oxygen in the surrounding environment during planktonic and biofilm growth (67). Additionally, data from a recent paper suggest that oral and lung *Prevotella* are more oxygen-tolerant than previously reported, with growth maintained in up to 5% oxygen and 24-h viability for subpopulations at atmospheric levels (68). Interestingly, the oxygen stress defense proteins associated with aerotolerance are less prevalent among the prominent gut *Prevotella* species (68), which were recently reclassified to new genera, including *Segatella copri* (formerly *Prevotella copri*) and *Leyella stercorea* (formerly *Prevotella stercorea*) based on multiple comparative genomic analyses. These findings are in alignment with another report comparing *Prevotella* isolate composition across the upper and lower airway, which identified the enrichment of select *Prevotella* isolates in the lower airway, suggestive of niche selection (66). Further studies are needed to determine the functional impact of *Prevotella* aerotolerance on colonization potential and *Prevotella*-host interactions. The highest density of *Prevotella* occurs in the upper respiratory tract, particularly in the oral cavity and oropharynx, with detection also noted in tonsillar tissue and the nasopharynx (66, 69, 70). Thus, while counterintuitive, the respiratory tract is an important mucosal site of residence for the anaerobe *Prevotella*.

The stability of *Prevotella* and the lung microbiome overall in healthy individuals remains an open debate. Under conditions in which airway clearance is impaired, such as cystic fibrosis (CF) and chronic obstructive pulmonary disease (COPD), alterations in microbial community composition are observed alongside a higher total microbial biomass, particularly in the lungs of people with CF (71–73). The microbiome in the lower airways of healthy individuals is more transient, with continuous aspiration of commensals from the upper airways and subsequent clearance via coughing, resident alveolar macrophages, and oscillation of respiratory cilia (74). In a longitudinal study on protected brush specimens from both healthy controls and patients with obstructive lung disease, the presence of a core taxa was noted alongside variation between samples collected at different times from the same subject (75), suggesting that while the microbiome in the lungs is more dynamic than in the intestinal tract, there appear to be core species that are consistently enriched in the lower airways of healthy individuals. Metabolomics and metagenomics studies may yield further insights into the contribution of live *Prevotella* activity and replication in the lower respiratory tract to *Prevotella*-mediated effects on lung homeostasis, as discussed below. Regardless of the stability of *Prevotella* in the lower airway, the lungs are frequently exposed to *Prevotella* based on the widespread detection and relatively high abundance of *Prevotella* DNA in the lungs, highlighting the importance of understanding how *Prevotella* exposures impact lung immune homeostasis.

## *PREVOTELLA* HETEROGENEITY WITHIN GENUS AND RESPIRATORY TRACT INFECTION ASSOCIATIONS

*Prevotella* genomes are highly dynamic, with an average of 50% of the total genes shared between any two genomes across different species (76), and only 17% of protein-coding genes comprising the core *Prevotella* genome (77). Over half of the original 55 *Prevotella* species were reclassified into four novel genera, including *Segatella*, *Leyella*, *Hoylesella*, and *Palleniella*, based on a 2022 study that included more recently isolated species in an updated analysis of the *Prevotella* genus (78). Within the remaining “true” *Prevotella*, comparative genomic analysis of *Prevotella intermedia* and *Prevotella nigrescens* isolates from health and periodontal disease demonstrated the high genetic plasticity of these species, with greater levels of genomic recombination in disease-derived isolates linked to carriage of more individual virulence factors such as secretion systems, proteinases, and toxins (79). These included genes encoding Bacteroides surface protein A (BspA), which mediates bacterial adherence and invasion, HlyB, involved in multidrug resistance, and the proteasome-associated ATPase (*mpa*), linked to pathogenesis (79). *P. intermedia* also expresses the protease interpain A, which inhibits complement by degrading complement factor 3 (80). The genetic diversity and plasticity within *Prevotella* complicate comparisons on the genus level regarding the impact of *Prevotella* in different disease contexts. From the available clinical literature, there appear to be select *Prevotella* species that are more strongly associated with infections, particularly periodontal abscesses, and may have more host tissue-damaging consequences. These species include *P. intermedia* and *P. nigrescens*, which are more frequently associated with periodontal disease and express proteases with toxicity to host cells (11, 81). Conversely, most other airway *Prevotella* species, including *Prevotella melaninogenica* and *Prevotella histicola*, are rarely associated with infection and typically negatively correlate with pathogen abundance and disease severity, as we discuss below. This dichotomy elevates the challenge of understanding how *Prevotella* influence respiratory tract homeostasis, as relationships described for one type or species may be reversed for others.

**Fig 1:**
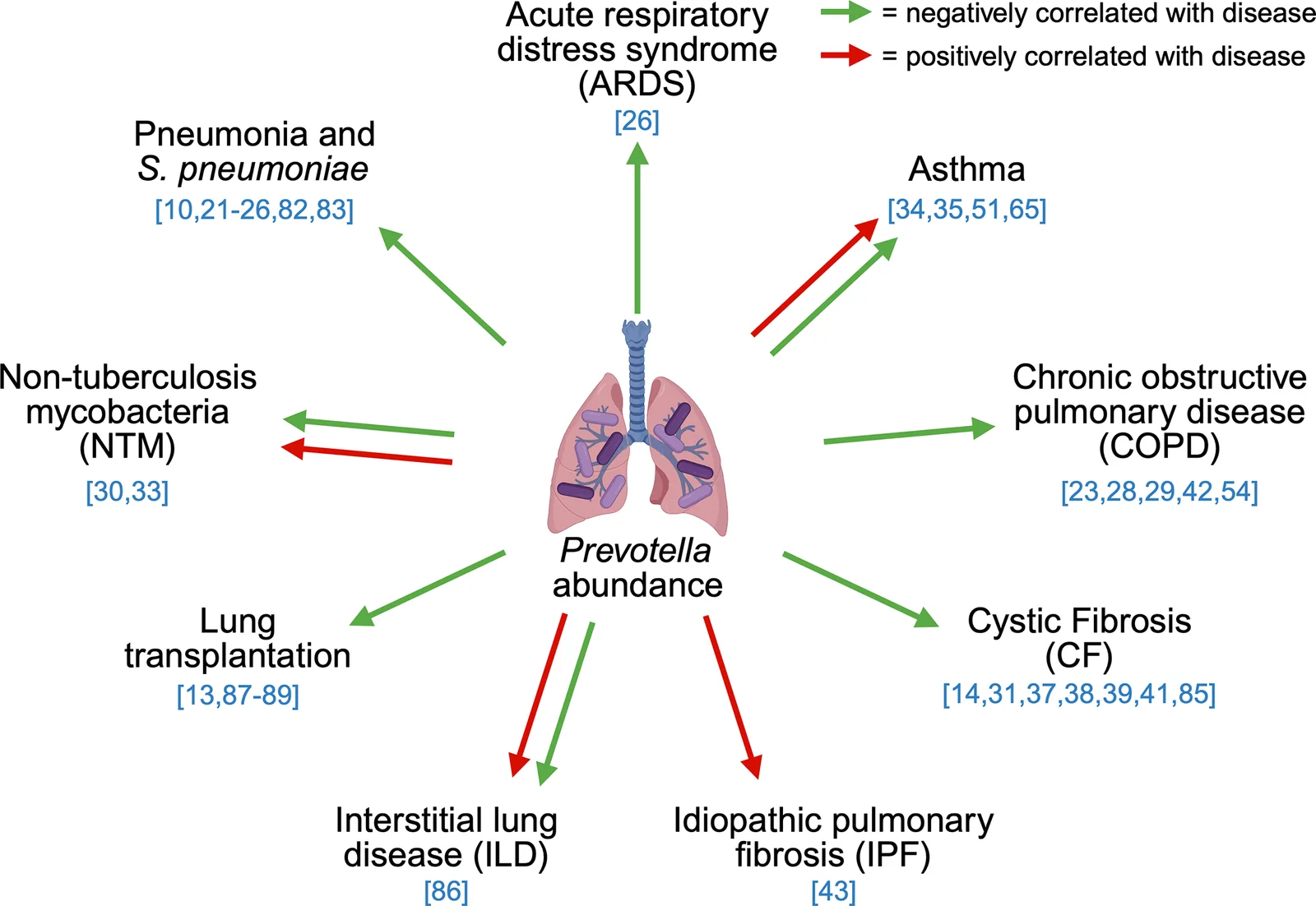
Lung *Prevotella* clinical correlations. The relative abundance of *Prevotella* in the lower airway is associated with lung infection and function in the context of chronic respiratory tract diseases. Negative (green) and positive (red) correlations between respiratory tract *Prevotella* abundance and lung infection or disease types are summarized (10, 13, 14, 21–26, 28–31, 33–39, 41–43, 51, 54, 65, 82, 83, 85–89).

While *Prevotella* are enriched in the healthy lungs, this relationship shifts in the context of pathogen infection, acute respiratory distress, and chronic lung diseases (Fig. 1). For example, *Prevotella* abundance is associated with reduced *S. pneumoniae* in both the upper and lower airway (10, 21–23). *Prevotella* are also reduced in the lungs of patients with community-acquired pneumonia, and respiratory tract *P. melaninogenica* was shown to be the most discriminative operational taxonomic unit (OTU) differentiating healthy adults from those with pneumococcal pneumonia (24, 25, 82). Furthermore, the onset of hospital-acquired pneumonia was associated with a loss of *Prevotella* and the presence of *Prevotella*-targeting bacteriophages (83). Similarly, in people with non-tuberculosis mycobacteria (NTM) pulmonary disease, successful NTM clearance was linked to an increased prevalence of *Prevotella* and higher lung microbiome diversity (33). In people without CF infected with *Pseudomonas aeruginosa*, *Prevotella* positively correlated with the pro-inflammatory cytokines TNF⍺, IL-17, and IL-6, as well as anti-inflammatory IL-10, although the impact of these responses as protective or detrimental was not evaluated (84). Across samples from 17 studies of critically ill patients with acute respiratory distress syndrome (ARDS) or hospital-acquired pneumonia, the relative abundance of *Prevotella* was significantly lower compared to healthy controls (26). These correlative studies are not sufficient to assign causality; however, there are consistent trends of less *Prevotella* in the context of infection and severe respiratory distress. Overall, dysbiotic microbiomes with lower diversity and a reduced relative abundance of *Prevotella* and other oral commensals correlated with significantly worse survival and longer recovery time in patients with acute respiratory failure (27). *Prevotella* and other obligate anaerobes declined over time in patients hospitalized with acute respiratory failure (32). In this setting, *Prevotella* inversely correlated with inflammatory markers including plasma soluble tumor necrosis factor receptor 1 (TNFR1) and receptor for advanced glycation end-products (RAGE) in the blood, as well as fractalkine (CX3CR1) in endotracheal aspirates, with a higher abundance of anaerobes correlating with significantly increased survival (32). In a separate cohort analyzed in the same study, *Prevotella* negatively correlated with the same plasma inflammatory markers in patients with severe COVID-19 (32). Together, these studies highlight a relationship between *Prevotella*, reduced lung and systemic inflammation, and improved survival compared to higher burdens of respiratory tract pathogens, which are linked to more robust inflammation and lower survival.

## *PREVOTELLA* ASSOCIATIONS WITH LUNG FUNCTION AND IMMUNE ACTIVATION DURING CHRONIC LUNG DISEASE

A recent systematic meta-analysis indicated that *Prevotella* were associated with lower concentrations of the pro-inflammatory cytokines IL-1β, IL-18, and TNFα across several studies of chronic lung diseases, correlating with improved lung function and reduced exacerbations (12). In one study, *Prevotella* was the genus that most strongly distinguished healthy individuals from those with COPD, in whom *Prevotella* was significantly depleted (23). COPD-like inflammation modeled through intranasal LPS and elastase exposures in mice significantly decreased *Prevotella* abundance in the murine lung, raising the question of whether lower levels of *Prevotella* are a risk factor for COPD or a consequence of chronic lung inflammation (50). In COPD patients treated with corticosteroids, *Prevotella* abundance was associated with significantly increased expression of tight junction genes, which promote lung barrier function (23). In the SPIROMICS study, *Prevotella* were significantly associated with several metabolites including adenosine monophosphate (AMP), adenosine, 5′ methylthioadenosine (MTA), and spermine, as well as reduced neutrophils, alongside better lung function and fewer symptoms in patients with COPD (54). Notably, other bacteria including *Streptococcus pneumoniae* were associated with lower lung function and a different set of metabolites (54). A significant correlation between *Prevotella* abundance and reduced neutrophils in COPD patients was also reported previously (28). In a SPIROMICS study follow-up, COPD patients with improved lung function exhibited *Prevotella* enrichment (29). Beyond the lungs, *Prevotella* abundance in the oral cavity also correlates with COPD outcomes. Lower levels of *P. melaninogenica* detected in saliva are also associated with a higher risk of severe COPD exacerbation (41). In COPD patients treated with pulmonary rehabilitation therapy, *Prevotella* increased in responders alongside reduced pro-inflammatory TNFα, IL-1β, and IL-18, in addition to anti-inflammatory IL-10, in saliva samples (41). As with infection and ARDS, this correlative evidence points to a relationship between lower *Prevotella* abundance in the lungs or oral cavity, higher inflammation, and more severe COPD.

The relationships between *Prevotella* and other chronic lung diseases are less clear, with mixed reports regarding a potentially beneficial versus disease-associated impact. This may indicate a more prominent role for *Prevotella* species-specific effects or that the same *Prevotella*-host interactions mediate different outcomes across intrinsically distinct conditions. For example, some studies report a significant reduction in *Prevotella* for people with asthma (34, 35), with species including *P. melaninogenica* correlating with reduced exacerbations and a lower percentage of granulocytes, including neutrophils and eosinophils (34). In contrast, another study reported *Prevotella* was associated with reduced lung function in people with asthma (51). *Prevotella* also correlated with more severe bronchiectasis in people with NTM infections, which were characterized as having elevated neutrophil extracellular traps (NETs) (30). For idiopathic pulmonary fibrosis (IPF), progression-free survival was linked to fibroblast responsiveness to toll-like receptor 9 (TLR9) activation, which negatively correlated with *Prevotella*, suggesting a detrimental immune modulatory association in this setting (42). This clinical finding is consistent with experimental evidence that *P. melaninogenica* worsened bleomycin-induced fibrosis in mice (43). Among patients with interstitial lung disease (ILD), a chronic lung disorder which includes IPF, individual *Prevotella* species had both positive and negative associations with inflammatory markers (86). For example, the *Prevotella* genus overall negatively correlated with the neutrophil-recruiting chemokine IL-8, indicating an immune suppressive effect, while the individual species *P. salivae* positively correlated with several pro-inflammatory responses, including C-reactive protein (CRP), IL-6, and TNFα (86). Collectively, these studies highlight circumstances including asthma and fibrotic lung disease as either benefiting or being exacerbated by *Prevotella*-host interactions, although the underlying mechanisms driving correlations between *Prevotella*, inflammation, and disease outcomes remain largely undefined.

*Prevotella* also have notable associations in lung transplant outcomes. In a recent study, *Prevotella* were the only bacteria in the donor lungs to correlate with better lung function in recipients (13). In lung transplant recipients, *Prevotella* were associated with a lower risk of allograft dysfunction and acute cellular rejection and reduced inflammatory cytokines in the lungs, with a longitudinal study suggesting that early enrichment with *Prevotella* and other oral microbes is critical for this beneficial association (87, 88). *Prevotella* have also been associated with lower levels of the inflammatory markers TNFα and cyclooxygenase-2 (COX2) in lung transplant patients, along with elevated markers of lung remodeling, including platelet-derived growth factor D (PDGFD) and the ratio of tissue inhibitor of metalloproteinase (TIMP) 1 to matrix metalloproteinase (MMP) 12 (89). Lower inflammatory markers paired with tissue remodeling are consistent with a pro-repair phenotype, which could be beneficial in the setting of lung transplant. Together, these findings suggest a protective phenotype for *Prevotella* in lung transplant outcomes. Overall, the inflammatory changes associated with *Prevotella* are highly context-dependent, highlighting the need for further research into the consequences of *Prevotella* immune modulation on lung health in various disease settings.

## *PREVOTELLA* IN THE CF LUNG: HOST RESPONSE AND INTERPLAY WITH OTHER MICROBES

In the lungs of people with cystic fibrosis (pwCF), there is a higher overall microbial burden as a direct result of defective mucociliary clearance (71). This community is dynamic, and detection of CF pathogens, including *S. aureus* and *Pseudomonas aeruginosa,* increases over time (71). Lung microbiome sequencing of explanted CF lungs compared to healthy donor lungs revealed that *Prevotella* were more abundant in healthy lungs compared to CF lungs in the context of severe CF lung dysfunction (37). However, *Prevotella* are an extremely prevalent and abundant member of the CF microbiome across the lifespan and are typically the most abundant obligate anaerobe, thriving in an environment with steep oxygen gradients due to mucociliary clearance dysfunction (14, 38). In pwCF, overall anaerobe abundance correlated with milder disease, including better lung function, in several culture-based studies (14, 31, 38). Sequencing studies have also associated *Prevotella* and/or oral anaerobes with higher lung function in pwCF (38, 39, 85), particularly for *P. melaninogenica* (40). Higher diversity microbiomes, which typically include anaerobes such as *Prevotella*, correlate with milder CF disease compared with lower diversity microbiomes, which are often dominated by CF pathogens (90–92). However, pulmonary exacerbations have been associated with increased anaerobes and fermentative metabolism (93), indicating that *Prevotella* are not always associated with superior lung function in pwCF.

It is unclear from these correlative studies whether *Prevotella* have a direct influence on lung function or pathogen persistence in the CF lungs. Both synergistic and antagonistic relationships have been identified between *Prevotella* and other common bacteria in the CF lungs, facilitated by a recent focus in the field on understanding the polymicrobial interactions underlying CF exacerbations and overall lung resilience in pwCF. Several groups have concentrated such efforts on defining interactions among a four-member community comprised of *P. melaninogenica*, *S. aureus*, *P. aeruginosa*, and *S. sanguinis*, identified as core members of the CF microbiome (52). In one study, most *Prevotella* isolates from the sputum of pwCF inhibited *P. aeruginosa* biofilm formation and protease secretion, suggesting a direct anti-pathogen effect, although strain and species-specific differences were noted (48). In this study, authors isolated up to three different *Prevotella* species per patient, indicating carriage of multiple *Prevotella* isolates in the CF lung. The *P. aeruginosa* biofilm-inhibitory effect was strongest for *P. intermedia* and *P. nigrescens* species, and cell-free culture supernatants were sufficient to reduce *P. aeruginosa* biofilms (48). While the supernatant factor(s) responsible were not identified, another group demonstrated that a *P. melaninogenica* glycoside hydrolase was sufficient to degrade *S. mutans* biofilms (57). *Prevotella*-pathogen interactions may also have negative consequences in pwCF, as *Prevotella* metabolism can support pathogen growth by cross-feeding. For example, *Prevotella* in a consortium of mucin-degrading bacteria increased growth of *P. aeruginosa* on mucin as a sole carbon source, although *Prevotella* alone was insufficient for this effect (58). Further research demonstrated that short-chain fatty acids (SCFAs), produced during the breakdown of mucin by *P. melaninogenica*, can be metabolized into acetate and succinate by *P. aeruginosa*. These metabolites subsequently promoted *P. melaninogenica* survival, suggesting a mutually beneficial intraspecies cross-feeding mechanism (53). Beta-lactamases expressed by *Prevotella* increased *P. aeruginosa* resistance to ceftazidime (94), which may indirectly promote *P. aeruginosa* survival. There are fewer studies analyzing *Prevotella* interactions with CF pathogens *in vivo*. However, in a non-CF mouse infection model, supernatants from *P. intermedia* were shown to exacerbate *S. aureus* infection and reduce neutrophil killing capacity (60). This effect was abrogated by modifying the *P. intermedia* supernatants through bacterial growth in subinhibitory concentrations of macrolide antibiotics, which was associated with a shift to lower ratios of neutrophils to alveolar macrophages and increased transcription of the pro-inflammatory genes *Ccr2*, *Tnf*α, and *Ifng* in lung tissue (95). *P. intermedia* supernatants also reduced *S. aureus* phagocytosis by neutrophils *in vitro* (60). Of note, *P. intermedia* supernatant increased levels of the major *S. aureus* toxin α-hemolysin *in vivo* but suppressed toxin expression *in vitro*, suggesting the elevated toxins detected during lung infection were due to immune escape facilitating the higher *S. aureus* burdens (60). In direct contrast to the effect of *P. intermedia* supernatants, heat-killed preparations of *P. melaninogenica* significantly improved clearance of *S. aureus* from the lungs of non-CF mice and increased lung neutrophil-mediated killing of *S. aureus* (44). Overall, the impact of *Prevotella* on lung function, inflammation, and exacerbations in pwCF may be intricately linked to positive and negative interactions with other CF microbes.

## *PREVOTELLA* AND ANAEROBE SPARING FOR CRITICALLY ILL PATIENTS

Despite anaerobes constituting only a small percentage of the causative agents of sepsis and pneumonia, anti-anaerobic antibiotics are still routinely administered empirically in combination with other broad-spectrum antibiotics (96). Longitudinal studies have shown that antibiotic therapy is associated with an increased risk of respiratory tract infections in the months following treatment (3, 6). Several recent studies have identified significant correlations between obligate anaerobes and pneumonia survival outcomes, which were affected by antibiotic use. First, reduced airway microbiome diversity was correlated with significantly lower survival in patients hospitalized with severe pneumonia (97). A defining feature of the lower diversity microbiomes was a reduction in oral commensal bacteria, many of which are obligate anaerobes such as *Prevotella*. Depletion of obligate anaerobic bacteria followed treatment with anti-anaerobic antibiotics or chronic oxygen therapy (97). Separately, two other studies, including one with ~16,000 patients, report increased patient mortality following anti-anaerobic antibiotic use (96, 98). During a U.S. shortage of piperacillin-tazobactam, a common empiric anti-anaerobic antibiotic, doctors began treating sepsis patients instead with cefepime, which has similar gram-negative coverage without anti-anaerobic activity. A retrospective cohort study that included this shortage period found that piperacillin-tazobactam was associated with an absolute mortality increase of 5% at 90 days (99), raising further questions about the role of anaerobes in protection against critical illnesses. The mounting evidence of detrimental effects from the widespread use of anti-anaerobic antibiotics, alongside limited clinical relevance for anaerobes in lung pathology, has issued calls for anaerobe-sparing in the treatment of critically ill patients (100, 101).

While antibiotic therapy impacts both the gut and airway microbiomes, both of which modulate lung homeostasis, a recent study identified oral and lung microbiota diversity as the strongest indicator of pneumonia outcomes, indicating a critical role for airway anaerobes in protection against pneumonia (32). Lung immune markers including IL-1α and IL-4 were also shown to correlate more with the composition of the lung microbiome than the oral or gut microbiota in a murine model (102). IL-1α is expressed by lung epithelial cells and alveolar macrophages (AMs) and is stimulated by microbial exposures, supporting neutrophil recruitment and innate immune response activation as a “frontline cytokine” in lung defense against bacterial pathogens. Collectively, these data suggest that respiratory tract anaerobes such as *Prevotella* are associated with a protective benefit in the context of pneumonia, and empiric treatment of infectious diseases including sepsis and pneumonia should shift toward anaerobe-sparing regimens.

## IMMUNE MODULATORY PROPERTIES OF *PREVOTELLA* IN THE RESPIRATORY TRACT

**Fig 2:**
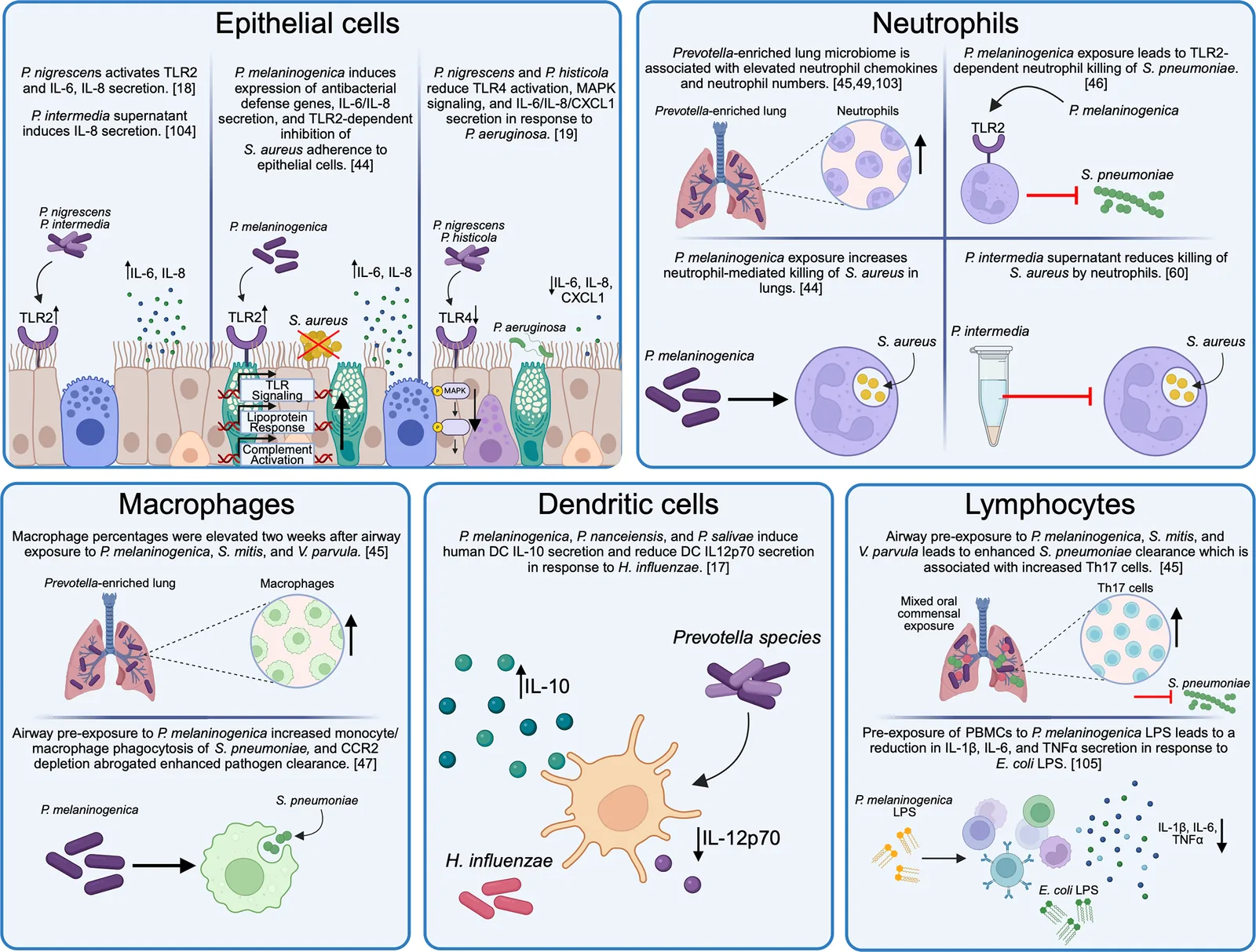
*Prevotella*-host interactions influencing epithelial and immune activation and defense against bacterial pathogens. Mechanistic studies have shown that several different types of host cells respond to *Prevotella* exposure. These responses include: the secretion of immune regulatory cytokines including IL-6, IL-8, CXCL1, and IL-10; modulation of lung macrophage, neutrophil, and Th17 cell populations; suppression of pathogen-induced inflammation and cytokine secretion; and direct effects on pathogen defense in epithelial cells (altered pathogen adherence) and neutrophils (altered pathogen killing) (17–19, 44–47, 49, 60, 103–105).

Beyond correlative studies, experimental evidence supports the notion that *Prevotella* contribute to lung immune tone or baseline activation status (Fig. 2). In two studies from Segal et al*.,* healthy individuals with a *Prevotella*-enriched lung microbiome “pneumotype” were reported to have subclinical inflammation including elevated neutrophil chemokines, a higher number of activated neutrophils, and greater Th17 cell frequencies in their lower airway (49, 103). Extending these findings, Wu et al. demonstrated that a single aspiration of *P. melaninogenica* in combination with two other oral commensals from the *Prevotella*-enriched pneumotype, *Streptococcus mitis* and *Veillonella parvula*, increased clearance of *S. pneumoniae* from the lungs of mice and improved survival 2 weeks after commensal exposure (45). At 2 weeks post-exposure to this microbe mixture, elevated percentages of lung CD4 and CD8 T cells, macrophages, and neutrophils were detected (45). Upon T cell subset analysis, the microbe mixture was shown to induce an increased percentage of IL-17-producing T cells in the lungs, and MyD88, an adaptor protein downstream of TLR signaling, was required for the increased Th17 response (45). These findings indicate that *P. melaninogenica*, in combination with *V. parvula* and *S. mitis*, increases the lung Th17 response associated with protection against pneumococcal pneumonia. In another study from this group, elevated percentages of neutrophils and Th17 cells were detected up to at least 2 months following exposure to the *P. melaninogenica*, *V. parvula*, and *S. mitis* mixture (30). In this case, the microbe mixture was shown to increase inflammation and NETs during NTM infection, which were associated with more severe bronchiectasis in people with NTM infections (30).

In a study from our group, lung exposure to *P. melaninogenica* alone led to rapid clearance of *S. pneumoniae* from the lungs and significantly improved survival (46). This protection was dependent on TLR2-mediated recruitment of neutrophils to the lungs and an enhanced capacity for recruited neutrophils to kill *S. pneumoniae* following *P. melaninogenica* exposure (46). In a follow-up study, we report that airway *Prevotella* exposure initiates a significant transcriptional shift away from an interferon-dominant response during *S. pneumoniae* infection toward a more antibacterial, pro-phagocytic profile in myeloid cells during *Prevotella*-enhanced pneumococcal clearance (47). This observation was associated with a functional increase in macrophage phagocytosis of *S. pneumoniae*, and recruited monocytes/macrophages were shown to contribute to improved infection defense in *Prevotella*-exposed mice (47). In separate work, we found that exposure to *P. melaninogenica* improved clearance of *S. aureus* from the lungs in a TLR2-dependent manner (44). In both cases, live or heat-killed *P. melaninogenica* were sufficient to improve pathogen clearance, underlining an important role for immune modulatory effects rather than direct pathogen interference in this setting. TLR2 activation was critical for *Prevotella*-induced protection against bacterial pneumonia, but treatment with a TLR2 agonist or *P. melaninogenica*-derived lipoproteins, which stimulate TLR2, was not sufficient to induce protection (44, 46), indicating a requirement for additional factors. In other work, gut-derived *S. copri* (formerly *P. copri*) were shown to directly increase survival of human blood neutrophils, which was associated with reduced expression of pro-apoptotic caspase-3 (106), indicating that *Prevotella* may enhance neutrophil survival as another mechanism contributing to improved pathogen defense.

A key component of *Prevotella*-induced protection against *S. pneumoniae* infection was the induction of the anti-inflammatory cytokine IL-10, which restrained infection-associated inflammation (46). *P. melaninogenica* and similar strains were sufficient to directly induce secretion of both TNFα and IL-10 by murine neutrophils *in vitro*, which was a TLR2-dependent response for protective strains, but not for *P. intermedia*, which failed to improve protection against *S. pneumoniae* and activated neutrophils regardless of TLR2 expression (46). A separate study indicated that *P. melaninogenica*, *P. nanceiensis*, and *P. salivae* can induce IL-10 secretion by human dendritic cells (DCs) and also reduced DC production of pro-inflammatory IL-12p70 by the pathogen non-typeable *Haemophilus influenzae (*17). Consistent with these findings, lung transplant recipients harboring a respiratory tract microbiome enriched for oral commensals including *Prevotella* had elevated levels of anti-inflammatory IL-10 (15). IL-10 was also correlated with *Prevotella* enrichment in people infected with *P. aeruginosa* (84). Pre-stimulation of murine peripheral blood mononuclear cells (PBMCs) with *P. melaninogenica* lipopolysaccharide (LPS) reduced secretion of pro-inflammatory cytokines including IL-6, IL-1β, and TNFα after subsequent exposure to *Escherichia coli* LPS (105). The suppressive effect of *Prevotella* LPS is due to the presence of penta-acylated lipid A, which is less immune stimulatory than hexa-acylated lipid A expressed by *E. coli* (107, 108). The LPS of *S. copri*, formerly *P. copri*, which is also under-acylated, was shown to be less cytotoxic to human monocytes compared to *E. coli* LPS (109). Together, this series of studies provides mechanistic evidence for *Prevotella* immune regulation in the lower airway, including the activation of innate and adaptive immune responses, which improve defense against bacterial pneumonia and restrain pathogen-associated inflammation.

## IMPLICATIONS OF *PREVOTELLA* METABOLIC ACTIVITY FOR HOST-BACTERIA INTERACTIONS

While the lung microbiome appears to be a more transient community than other sites, recent studies suggest a metabolic capacity for *Prevotella* in the lower airways. In the gut, *Prevotella* and their close relatives are important producers of short-chain fatty acids (SCFAs) including acetic acid, butyric acid, and valeric acid (59). SCFAs have both immune stimulatory and suppressive effects, including redirecting or amplifying TLR responses (110–112). Prominent respiratory tract species including *P. melaninogenica* also produce SCFAs (59). The SCFAs butyrate and propionate detected in lung epithelial lining fluid of human immunodeficiency virus (HIV) + subjects on antiretroviral therapy (ART) were associated with an enrichment in oral commensals including *Prevotella* (113). In the CF lung, where bacterial loads are higher than in health, SCFAs are detected at 1–40 μM in bronchoalveolar lavage (BAL) fluid, with acetic acid measured at the highest levels (59). Anaerobes including *Prevotella* can degrade mucins into amino acids and SCFAs, making the high levels of mucin present in the sputum of pwCF a rich substrate for *Prevotella*-derived SCFAs (58). In a non-CF cohort, BAL levels of SCFAs were lower, with the greatest levels detected for acetate, at ~10 μM, while only some subjects had detectable levels of propionate, isovalerate, and butyrate (55). Upper airway levels of SCFAs were 10–100 times higher; however, there was no correlation between upper and lower airway concentrations of individual SCFAs, suggesting that BAL levels were not solely a consequence of upper airway aspirations (55). *Prevotella*-derived SCFAs are an important mechanism for gut *Prevotella* regulation of the intestinal mucosal barrier and lung infection outcomes (114). However, much of the mechanistic work linking gut *Prevotella* to barrier function and immune homeostasis was completed in *S. copri*, formerly *P. copri,* which was classified into a separate genus based on changes including distinct carbohydrate-active enzyme (CAZyme) profiles compared to “true” *Prevotella* species enriched in the airway (115). These include enzymes required for mucin degradation and SCFA production, making it unclear whether lessons learned from gut *Segatella* regulation of mucosal barrier function and immune homeostasis translate to the lungs and the impact of airway *Prevotella* species. Beyond SCFAs, the lower airway contains differentially enriched metabolites compared to the upper airway, many of which impact immune function and are made, modified, or used by airway bacteria (56). Microbiome and metabolomic analyses identified a correlation between *Prevotella* enrichment and higher adenosine and polyamine levels in the lower airways, which are linked to slower lung function decline and milder COPD symptoms (54).

Emerging evidence connects lower airway metabolic changes with bacterial activity based on transcriptomic analysis. In lower airway samples from healthy adults who had detectable levels of *Prevotella* and other oral commensals by 16S rRNA sequencing, RNA metatranscriptomic sequencing was consistent with active microbial metabolism (55). In a follow-up study, metagenomic and metatranscriptomic analyses predicted increased functional activity of *P. melaninogenica* in the lower airways, compared to the upper airways, despite being less abundant in the former (56). *Prevotella* was predicted to be a top contributor to several metabolic pathways (56). To validate these findings, Wong et al. demonstrated that murine lung colonization with *P. melaninogenica*, *V. parvula*, and *S. mitis* led to regional enrichment of metabolites in the lower airways, including methionine and tyrosine (56). Furthermore, ^13^C isotope labeling experiments demonstrated the ability of *P. melaninogenica* to produce specific metabolites in the lungs, including adenosine, inosine, and glutamate (56), which exhibit immunomodulatory properties (116–118). As previously discussed, adenosine was correlated with *Prevotella* enrichment and lung function (measured as FEV_1_) in a COPD cohort (54) and plays a role in airway surface liquid volume regulation (118). Further studies are needed to understand whether metabolites specifically produced by *Prevotella* significantly impact lung immune tone.

## *PREVOTELLA* ACTIVATION IN THE RESPIRATORY TRACT EPITHELIUM

The lung epithelium serves as a critical first line of defense by maintaining barrier integrity and modulating the immune response following exposure to different stimuli. In addition to immune cells, *Prevotella* stimulates CD45-negative cells from the lungs, which include respiratory tract epithelial cells (17, 119). In the CFBE41o− (F508del) cell line, which is derived from bronchial epithelial cells of a pwCF, Bertelsen et al. demonstrated that *P. nigrescens* activates TLR2 and an NFkB-driven inflammatory response, including increased expression of IL-6 and IL-8, although this response was more subdued compared to cytokine induction by the pathogen *P. aeruginosa* (18). *P. melaninogenica* also induced IL-8 production in CFBE41o− epithelial cells (59). Of note, TLR activation in CFBE41o− cells was species-specific, as *P. histicola* activated both TLR2 and TLR5, while *P. nigrescens* activated TLR2 but not TLR5. IL-6 and p65 DNA binding were both TLR2-dependent, as these were reduced with TLR2 siRNA knockdown (18). Epithelial cell responses to *Prevotella* species vary based on the presence of functional CFTR. In comparisons between the CFTR mutant CFBE41o− epithelial cells and the wild-type cell line 16HBE41o−, derived from a different donor, *P. melaninogenica*-induced IL-8 was significantly higher in the CFTR mutant cells (59). In contrast, isogenic CFTR-corrected cells had higher levels of IL-6 and IL-8 production in response to *P. melaninogenica* than CFTR-deficient CFBE41o− cells (44). In this case, the lower cytokine response in CFBE41o− cells was rescued by CFTR modulator therapy (44), suggesting that restoration of CFTR function improves the epithelial response to *Prevotella*. In the same study, *P. melaninogenica* regulation of epithelial cells was analyzed by RNA sequencing. In CFTR-corrected (wild-type) epithelial cells, *P. melaninogenica* exposure induced several antibacterial defense response pathways, including TLR signaling, lipoprotein response, and complement activation (44), all of which were significantly lower in CFTR mutant CFBE41o− cells. Outside CFTR mutant epithelial cells, *P. intermedia* supernatants were shown to induce IL-8 production in the wild-type lung epithelial cell line NCI-H292 (104). Human bronchial epithelial cells stimulated with *P. bivia* supernatants experienced changes in cell lipid metabolism, which holds implications for lung inflammation attributed to asthma (36). Together, these studies suggest that *Prevotella* interacts with epithelial cells by inducing TLR signaling (primarily TLR2), propagating responses including antimicrobial defense genes and secretion of pro-inflammatory cytokines and chemokines.

*Prevotella* interactions with respiratory tract epithelial cells may play an important role in regulating the epithelial response to infections and insults contributing to lung injury. For example, in a diesel-exhaust-induced lung injury model, murine lung epithelial cells treated with *P. nanceiensis*-derived extracellular vesicles had attenuated transcription of pro-inflammatory genes including *Tnf*α, *Il6*, and *Cxcl1*, and lower neutrophil activation (16). Several other studies have investigated how *Prevotella* exposures impact bacterial pathogen interactions with epithelial cells. *P. intermedia* supernatants were shown to increase A549 alveolar epithelial cell expression of the gene encoding platelet-activating factor receptor (PAFR), contributing to higher *Streptococcus pneumoniae* epithelial adherence (61). In contrast, we found that epithelial exposure to live or heat-killed *P. melaninogenica* reduced *S. aureus* adherence (44). As with *P. melaninogenica*-induced cytokine stimulation, this effect was TLR2-dependent (44), suggesting that species-specific *Prevotella* regulation of epithelial cells contributes to altered pathogen adherence. *Prevotella* exposure also modifies pathogen-induced inflammation in epithelial cells, as noted for the effect in the epithelial injury model above. In CFTR mutant CFBE41o− cells, the addition of *P. nigrescens* or *P. histicola* after the CF pathogen *P. aeruginosa* significantly reduced TLR4 expression and MAPK signaling, resulting in decreased secretion of the cytokine IL-6 and neutrophil recruiting chemokines IL-8 and CXCL1 (19). Spent media from these *Prevotella* strains were sufficient to suppress *P. aeruginosa*-induced epithelial responses and directly impair *P. aeruginosa* growth (19). Similarly, several *Prevotella* species isolated from pwCF reduced NFkB expression in response to *P. aeruginosa* lipopolysaccharide (LPS) in a 3D lung epithelial model (20). These insights indicate that epithelial exposures to airway *Prevotella*, which are common throughout the respiratory tract, influence epithelial-derived cytokines and chemokines that regulate the lung immune response both at baseline and in the context of pathogen infection. There are also epithelial-intrinsic changes following *Prevotella* exposure, which impact pathogen adherence directly, highlighting *Prevotella* interactions with the respiratory tract epithelium as a potential critical modulator of pathogen uptake and infection.

## LEVERAGING THE AIRWAY MICROBIOME FOR IMPROVED HEALTH: KEY KNOWLEDGE GAPS

While significant progress has been made toward our understanding of how *Prevotella* impacts respiratory tract health, including lung infection defense, several key knowledge gaps must be addressed to leverage these insights for clinical intervention (Table 2).

**TABLE 2:** Key knowledge gaps in airway *Prevotella*-host interactions

Topic	Knowledge gap
Regulation of immune homeostasis by *Prevotella* at distinct mucosal sites	What concepts regarding gut *Prevotella/Segatella* regulation of intestinal barrier function and immune activation translate to airway *Prevotella* in the lung?
*Prevotella* species and isolate specificity governing host cell activation	What are the defining features driving divergent effects for different *Prevotella* species and isolates on host activation and disease outcomes?
The disease-specific cues for beneficial versus harmful *Prevotella* effects	When are *Prevotella* immune interactions beneficial versus harmful, and how do these differ by baseline lung function and disease?
*Prevotella* in the broader microbial community	*Prevotella* are active members of a broader airway microbial community; what are the synergistic and antagonistic relationships with other bacteria contributing to *Prevotella-*host effects?

Immunomodulatory effects of individual *Prevotella* species have been characterized to some extent, and *P. melaninogenica* has been studied in combination with oral commensals of different genera (45). Besides *P. melaninogenica*, other *Prevotella* species have been shown to mediate TLR2-dependent protection from acute pneumococcal pneumonia, including *P. tannerae*, *P. buccae*, and *P. nanceiensis* (46). However, no studies have examined how a community of mixed *Prevotella* species impact lung immune tone and how varying the ratios of these species may shift host immune responses. Another important question is the dose and exposure frequency required for *Prevotella*-mediated effects on lung immune homeostasis and infection defense. A previous study from our group determined that 10^5^ CFUs of live *P. melaninogenica* was the minimum dose needed to induce enhanced clearance of *S. pneumoniae* 24 h later (46), and *Prevotella* burdens of 10^5^ CFUs/mL have been reported in human bronchial alveolar lavage samples (15). Further research is needed to understand how long a single airway exposure of *P. melaninogenica* confers protection against pneumococcal pneumonia, and how repeated commensal exposures, akin to repeated aspirations in healthy people, affect lung immune tone and subsequent protection against pneumonia.

The critical immune modulatory features of *Prevotella* remain incompletely defined. *P. melaninogenica* treatment with lipoprotein lipase diminished protective clearance of *S. pneumoniae*; however, isolated lipoproteins were insufficient to induce the protective response (46). Identification of the other *P. melaninogenica* factors required to mediate this protective response in combination with lipoproteins could facilitate development of immunomodulatory therapeutics derived from specific *Prevotella* components. Further characterization of the immune pathways induced by *Prevotella* for protection, particularly those beyond TLR2, could also enable formulation of host-targeted therapeutics against respiratory pathogens. Others have shown that combined treatment with TLR2/6 and TLR9 agonists in mice improved survival from several gram-positive and gram-negative bacterial lung infections (120), as well as influenza A pneumonia (121). Administration of flagellin, a TLR5 agonist, in combination with antibiotic treatment led to increased neutrophil influx to the lungs, decreased bacterial load, and increased survival of *S. pneumoniae*-infected mice, compared to treatment with antibiotics alone (122, 123). Identifying the critical host cells and signaling pathways induced by immunomodulatory commensals such as *Prevotella* would support the development of immune-targeted therapeutics for lung infection and chronic lung disease.

Studies in animal models and clinical trials have demonstrated the therapeutic capacity of inhaled probiotics. An inhaled live biotherapeutic comprising multiple live *Lactobacillus* strains attenuated pulmonary neutrophil influx and tissue damage in murine models of bronchopulmonary dysplasia (BPD) and COPD, indicating the potential for inhaled probiotics to slow progression of chronic lung diseases (124). Several clinical trials examining the protective effects of inhaled probiotics against respiratory pathogens have been performed with the oral commensal *Streptococcus salivarius*. In a 4-month-long clinical trial that followed 112 participants, those who strictly adhered to a daily dose of inhaled *S. salivarius* strain K12 experienced more days free from upper respiratory tract infections (URTIs) compared to those in the placebo group (125). In another trial, a combination of *S. salivarius* 24SMB and *Streptococcus oralis* 89a was administered nasally twice daily for 90 days to children with recurrent URTIs, leading to a 64.3% reduction in URTIs (126). Further research is needed to define the long-term safety and efficacy of inhaled probiotics, including the potential use of *Prevotella* as an aerosolized probiotic, which has not yet been evaluated.

While studies investigating the therapeutic potential of *Prevotella* airway exposures are limited, a series of studies have demonstrated that gut exposure to *P. histicola*, which is also found in the lower airway, has anti-inflammatory properties in mice, including delayed onset of type 1 diabetes in non-obese diabetic (NOD) mice (127), suppression of experimental autoimmune encephalomyelitis (EAE) disease (128–130), and, unlike *S. copri*, reduced rheumatoid arthritis (RA) severity (131). However, *P. melaninogenica* was not protective against RA, and only had a partial effect against EAE (129, 131). These studies highlight species diversity regarding the impact of gut-exposed *Prevotella* on systemic immune regulation, and it is not clear how gut *Prevotella* exposures compare to airway *Prevotella*-mediated effects on lung immune homeostasis. In the gut, both *P. melaninogenica* and *S. copri* increased colonic Th17 cells and elevated systemic levels of cytokines including IL-6, TNFα, and IL-10 (132), which is similar to the cytokine profile induced following lung exposure to *P. melaninogenica* (46), although these were not directly compared.

While progress has been made in delineating correlations between *Prevotella* and lung health and disease, most clinical studies rely on genus-level data to report *Prevotella* associations. Further resolution of the human lung microbiome at the species level will aid in identifying specific *Prevotella* species associated with pulmonary health and immune modulation. Such work could help to resolve opposing findings of *Prevotella*-disease correlations between studies, which may be attributed to differential species enrichment. Additionally, few studies have examined the impact of individual *Prevotella* species in murine airway disease models. Such analysis would inform, at the species level, the precise immune mechanisms influenced by *Prevotella* exposure and the differential impact of *Prevotella* species on airway diseases, as suggested by the clinical literature. Species-level studies could also serve to accelerate the development of probiotic-based therapeutics for the prevention or treatment of airway disease.

In summary, the application of *Prevotella* as a therapeutic strategy will benefit from further studies elucidating how *Prevotella*-host interactions dictate altered lung immunity and function. Current knowledge regarding *Prevotella*-associated benefits suggests clinically actionable interventions, such as the use of anaerobe-sparing antibiotic regimens, to improve survival in critically ill patients. The current challenge for the field is to generate a greater depth of understanding regarding how *Prevotella* regulate lung immune activation and overall health, including the impact of different *Prevotella* species and isolates on individual and integrated cellular immune response pathways, and the intersection of these *Prevotella*-host interactions with other microbe-host interactions throughout the respiratory tract.
